# Brief Report: The Role of Task Support in the Spatial and Temporal Source Memory of Adults with Autism Spectrum Disorder

**DOI:** 10.1007/s10803-015-2378-9

**Published:** 2015-02-19

**Authors:** Dermot M. Bowler, Sebastian B. Gaigg, John M. Gardiner

**Affiliations:** Autism Research Group, Department of Psychology, City University London, Northampton Square, London, EC1V 0HB UK

**Keywords:** Autism, Memory, Spatial source, Temporal source, Task support

## Abstract

Adults with autism spectrum disorder (ASD) show intact recognition (supported procedure) but impaired recall (unsupported procedure) of incidentally-encoded context. Because this has not been demonstrated for temporal source, we compared the temporal and spatial source memory of adults with ASD and verbally matched typical adults. Because of difficulties with temporal processing in ASD, we predicted ASD adults would benefit from test support for location but not temporal occurrence of studied words. We found similar levels of recognition and source memory for both groups but there was a greater effect of support on memory for location source in the ASD group. The lack of an effect of support for temporal source may simply reflect a difficulty in operationalising temporal cues.

## Introduction

Individuals with autism spectrum disorder (ASD) are now known to have subtle but characteristic difficulties with memory (see Boucher and Bowler 2008; Boucher et al. 2012, for reviews). Relatively intact performance is seen on tasks, such as cued recall or recognition, where the test procedure provides clues to the learned material (Bowler et al. 2000), but tasks such as free recall, which provide fewer such cues, often reveal deficits although in higher-functioning individuals although these difficulties are often seen only on more complex or multi-trial tasks (Bowler et al. 2008, 2010; Cheung et al. 2010; Williams et al. 2006). The difference in performance between supported and unsupported tasks led Bowler, Matthews and Gardiner (1997) to formulate a *Task Support Hypothesis* (TSH) of memory in ASD, which stated that memory performance in this group would be superior on tasks that provided more support for the studied material at test. The TSH has proved useful in helping to resolve conflicting findings in the source memory literature. Bowler et al. (2004) noted that some studies showing impaired source memory, such as that by Bennetto et al. (1996), utilized unsupported test procedures, whereas those that showed intact source memory (such as Farrant et al. 1998) utilized supported procedures. In a study that systematically manipulated the test procedures in a source memory task, Bowler et al. (2004) demonstrated that recognition by individuals with ASD of studied item location, voice of presentation and item-related actions was as good as that of comparison participants. Their recall of this information, however, was diminished. By showing that supported test procedures such as recognition enhanced source memory, Bowler et al. (2004) extended TSH to source memory.

Source memory difficulties in ASD are thought to reflect broader difficulties with *episodic memory* (Bowler et al. 2000, 2007), which involves not only the reconstruction of the context of a memory as well as what Tulving (2001) called *mental time travel*, which involves the self mentally travelling back to a re-creation of the recollected episode. Lind and Bowler (2010) recently extended these findings to *Episodic Future Thinking*, which is the ability to project oneself into plausible future situations. Both episodic memory and episodic future thinking rely on a sense of the temporal order of events, and disturbances in these functions imply difficulties with the processing of temporal order. People with ASD are known to make more temporal order errors in serial recall tasks (Poirier et al. 2011) and also on serial order reconstruction tasks that minimise demands on item retrieval (Gaigg et al. 2014), suggesting that general retrieval support may not be sufficient to facilitate the retrieval of temporal source information. When test procedures provide more explicit temporal cues, by contrast, individuals with ASD benefit considerably. Thus, a recent finding by Williams, Boucher, Lind et al. (2012) reports that children with ASD performed better on event-based compared to time-based prospective memory tasks. The former task signals to the participant that a certain period of time has elapsed, and that they must then perform a given act; the latter requires participants to estimate when a given period of time has elapsed, without having any external cue. As such, the two tasks can be considered to be supported and unsupported time estimation tasks.

Given that the non-temporal source memory of individuals with ASD has been shown to be enhanced by task support, the present study was designed to test whether or not the provision of task support would be as effective in enhancing memory for temporally defined incidentally encoded information. We used a test procedure that was as similar as possible across the temporal and non-temporal tasks by testing whether or not temporal source memory would be facilitated when support is provided in the form of explicit labels that identify particular periods within a longer episode or that identify particular spatial locations. The former was chosen because such labels are frequently used in every-day life (e.g., Where did you go *first*?); the latter because it replicates procedures used in earlier research (e.g. Bowler et al. 2004) On the basis that individuals with ASD have difficulties with diachronic thinking (envisaging events as unfolding over time, Boucher et al. 2007), temporal estimation (Martin et al. 2010), time perception (Allman et al. 2011) and time-based prospective memory (Williams et al. 2012), we hypothesised that task support would be as effective in improving recognition of spatially-defined, incidentally-encoded context but less effective in enhancing recognition of temporally-defined incidentally-encoded context in high functioning adults with ASD.

## Method

### Participants

Eighteen verbal individuals with ASD (5 female, 13 male) and 18 typical individuals (4 female, 14 male) participated. Groups were closely matched in terms of chronological age and cognitive ability measured by the Wechsler Adult Intelligence Scale (WAIS-III^UK^; The Psychological Corporation, 2000, see Table 1). Participants with ASD were recruited from a panel maintained by the Autism Research Group at City University London. A review of medical records confirmed that they had all received their diagnosis according to DSM-IV-TR (American Psychiatric Association 2000) criteria by experienced clinicians within the National Health Service (NHS). Assessment with the Autism Diagnostic Observation Schedule (ADOS; Lord et al. 1989) by suitably trained individuals further corroborated difficulties in reciprocal social and communicative behaviours that are the hallmark of the disorder for 15 of the 18 participants. For logistical reasons, the ADOS could not be administered to the remaining three participants. The typical comparison participants were recruited via local newspaper advertisements. All were free of psychotropic medication and did not report any family history of neuropathology or psychiatric illness.

**Table 1:** Psychometric characteristics of the ASD and typical comparison group

Measure	ASD (*n* = 18)			Typical (*n* = 18)		
	*M*	*Range*	*SD*	*M*	*Range*	*SD*
Age (years)	36.0	18–58	13.5	33.6	18–57	11.5
VIQ^a^	106.1	81–144	16.8	106.8	84–138	16.4
PIQ^b^	106.3	77–155	20.7	105.5	72–134	15.6
FIQ^c^	107.2	80–155	20.5	106.6	84–140	16.4
ADOS^d^ Com	2.9	0–6	1.6	–	–	–
ADOS^d^ RSI	6.9	3–12	2.9	–	–	–
ADOS^d^ Tot	9.7	5–16	3.4	–	–	–

^a^Verbal IQ (WAIS-R^UK^ or WAIS-III^UK^)

^b^Performance IQ (WAIS-R^UK^ or WAIS-III^UK^); scores for two TD participants were not available

^c^Full-Scale IQ (WAIS-R^UK^ or WAIS-III^UK^); the score for two TD participants was not available

^d^Autism Diagnostic Observation Schedule Communication (Com), reciprocal social interaction (RSI) and total (Tot) algorithm scores; ADOS scores for three ASD participants were not available

### Materials and Design

Study lists consisted of 27 words presented in three temporally distinct lists of nine words. All words were selected from the University of Western Australia MRC Psycholinguistic Database (Coltheart 1981), They were concrete 5–6 letter long nouns of an average written frequency of 30 per million (*SD* = 15) (Kucera and Francis 1967) and average Concreteness ratings of 585 (*SD* = 35) on an arbitrary 100–700 scale (see Coltheart 1981). Groups of nine words were randomly assembled with the constraint that they closely matched in terms of average letter length, word frequency and concreteness ratings, whilst at the same time avoiding obvious semantic and/or categorical associations between words. These groups of words were systematically counterbalanced across study blocks and screen locations such that each word appeared equally often in each block and each screen position across subjects. For the recognition test, an additional 18 words selected on the same basis served as lures and across subjects lures and to-be-remembered words were also counterbalanced such that the lures for half of the participants were part of the to-be-remembered set of words for the other and vice versa.

### Procedure

Words were presented in three temporally distinct lists. Each list was explicitly labelled as the 1st List, 2nd List and 3rd List, and these labels were presented on the screen for 6 s before the words appeared. Within each list, each word appeared for 4 s in black Arial font (size 48) either at the Top, the Middle or the Bottom of the screen within a rectangular black frame. The test phase, which followed a short (~5 min) unrelated non-verbal distracter task (mental rotation or number matching), presented participants with studied words and lures in a ‘neutral’ screen position (approximately half way between the Top and Middle positions of the study phase). Participants were asked to indicate whether or not they recognised the word from as one from the study list. If they did not, the next word was presented but if participants did recognise a word they were asked either an unsupported source recognition question (‘on which list did you see the word/where did the word appear) or a supported recognition question where the explicit list labels and rectangular location frames were presented on the screen for participants to choose from. If participants indicated that they could not remember the source, they were asked to guess. Because it could not be anticipated which or how many of the study words would be recognised at test, the four source memory questions were selected pseudo-randomly at test to ensure that they were distributed as evenly as possible across the various stimulus conditions. The whole experiment lasted about 10–15 min.

## Results

**Fig. 1:**
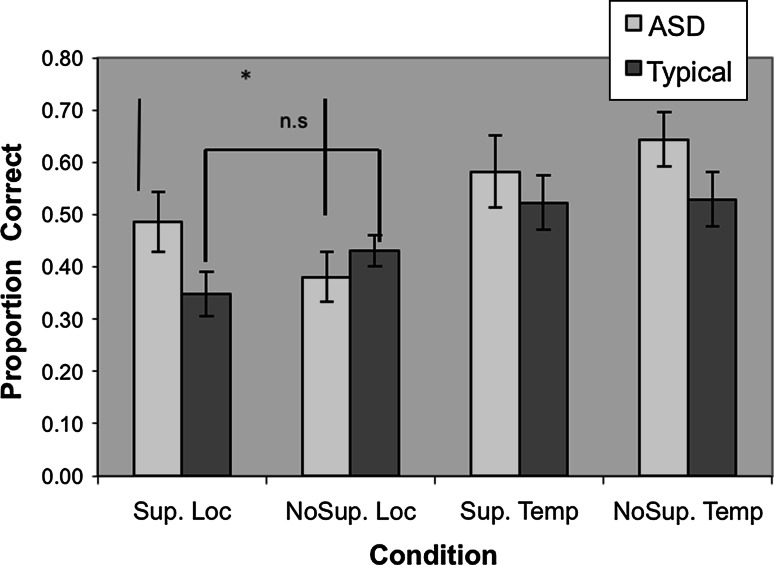
Supported and unsupported temporal and location source for the two groups. *Significant difference (*p* < .05). *Sup. Loc* supported location, *NoSup. Loc* non supported location, *Sup. Temp* supported temporal, *NoSup Temp* non supported temporal

Analysis of corrected hit rates (hits-false alarms) revealed no significant difference in overall recognition between groups (ASD: *M* = 0.65 *SD* = 0.23, Comparison: *M* = 0.68, *SD* = 0.19, *t* = 0.41, *df* = 34, *ns; Cohen*’*s d* = 0.14). Source memory was determined by the number of correct source identifications as a proportion of the number of recognition hits. Scores for supported and unsupported temporal and location sources are set out in Fig. 1. Analysis of these data by means of a 2 (Group) × 2 (Source Type) × 2 (Support) mixed, repeated measures ANOVA, revealed a significant main effect for Source Type, with superior temporal over location source (*F*(1,34), = 21.33, *p* < .01, effect size *r* = 0.62; Temporal Source *M* = 57, *SD* = 0.20; Location Source *M* = 0.41, *SD* = 0.16.), and a significant Group by Source Type by Support interaction (*F*(1,34) = 4.81, *p* < .04, effect size *r* = 0.35). The interaction is illustrated in Fig. 1. Planned *t*-tests, comparing groups on the difference between supported and unsupported location and temporal source trials, showed that the ASD group benefited significantly more from support on the location source trials than did TD participants (*t* = 2.66, *df* = 34, *p* < .02, *Cohen*’*s d* = 0.90; *ASD* *M* = 0.11, *SD* = 0.24; *TD* *M* = −0.08, *SD* = 0.18), whereas on the temporal source trials, groups did not differ in their sensitivity to the support manipulation and, in fact, did not appear to benefit greatly from support (*t* = 0.60, *df* = 34, *ns*, *Cohen*’*s d* = 0.20; *ASD* *M* = −0.06, *SD* = 0.25; *TD* *M* = −0.01, *SD* = 0.30).

## Discussion

The finding that source memory for location in participants with ASD benefitted from the provision of support at test replicates the findings of Bowler et al. (2004) and provides further corroboration of the TSH in ASD. But the most important finding of the present study is the failure to find any effect of support on temporal source memory for either of the groups, nor any overall group difference in temporal source memory. Inspection of Fig. 1 shows that provision of support neither helped nor hindered the performance of either group: the typical participants showed identical levels of performance on both supported and unsupported temporal source conditions and the ASD group showed a non-significant increase in the supported condition. These observations indicate that for the typical group, this absence of an effect of support is in line with the observations of Bowler et al. (2004) and suggests that presence or absence of support is less of an issue for typical individuals. For the ASD group, however, the contrast between the positive effect of support on spatial source and the absence of its effect on temporal source are consistent with the idea that individuals with ASD have specific difficulties in time processing. This is evidenced by their poorer performance compared to typical individuals on temporally-loaded tasks such as serial recall (Poirier et al. 2011) or diachronic thinking (Boucher et al. 2007). An alternative explanation of the current findings hinges on the intrinsically ephemeral nature of temporal phenomena and the consequent difficulty in operationalising an adequate representation of different time periods to use as support at test. Whereas the position of stimuli presented at the top, middle or bottom of a screen can easily be represented at test by markers in those positions, stimuli presented in the 1st, 2nd or 3rd block of trials can only be labelled using the words ‘Block 1’ etc., and, moreover, need to be placed in spatial positions about the screen. It could be argued that someone with a temporal processing difficulty would find it hard to grasp the correspondence between the labels and the temporal occurrence of events in a way that someone without such difficulties would not. Unlike location source tasks, on which individuals with ASD benefit from the provision of support, temporal source tasks have an added level of complexity that appears to render them more resistant to task support. Future studies should be directed at unpacking this complexity in order to determine whether the difficulty reported here is a function of difficulties understanding temporal order *per se*, or a difficulty in learning to associate temporal order with ordering in another dimension, either spatial (left–right organisation on a screen) or symbolic (labels ‘first’, ‘second’ etc.). On the basis of the current study’s observation of a difference in effectiveness between support for temporal and non-temporal source, designers of supported environments and educational settings should be vigilant in trying to organise instructional materials spatially (‘what was at the top/bottom?) rather than temporally (‘what came first?). And when providing support for source memory, instructors should avoid inadvertently taxing memory for temporal source, for example by asking a child to remember whether something was ‘first’, ‘second’ or ‘third’ with those labels presented from left to right. This creates the impression that the support is visuo-spatial when the underlying memory is in fact temporal.

The present results confirm the view that individuals with ASD rely to a greater extent than typical individuals on support at test for non-temporal source. The findings also place a constraint on the TSH as a general principle of memory function in the ASD population, limiting it to non-temporal source. The failure to find a support effect for temporal source may simply be a reflection of the difficulty of implementing adequate support for temporal aspects of material, or may reflect an underlying difficulty in processing temporal information.

## References

[CR1] Allman MJ, DeLeon IG, Wearden JH (2011). Psychophysical assessment of timing in individuals with autism. American Journal of Intellectual and Developmental Disabilities.

[CR2] American Psychiatric Association (2000). Diagnostic and statistical manual of mental disorders, fourth edition—Text revision.

[CR3] Bennetto L, Pennington BF, Rogers SJ (1996). Intact and impaired memory function in autism. Child Development.

[CR4] Boucher J, Bowler D (2008). Memory in autism: Theory and evidence.

[CR5] Boucher J, Mayes A, Bigham S (2012). Memory in autistic spectrum disorders. Psychological Bulletin.

[CR6] Boucher J, Pons F, Lind S, Williams D (2007). Temporal cognition in children with autistic spectrum disorders: Tests of diachronic thinking. Journal of Autism and Developmental Disorders.

[CR7] Bowler DM, Gaigg SB, Gardiner JM (2008). Subjective organisation in the free recall of adults with Asperger’s syndrome. Journal of Autism and Developmental Disorders.

[CR8] Bowler DM, Gaigg SB, Gardiner JM (2010). Multiple list learning in adults with autism spectrum disorder: Parallels with frontal lobe damage or further evidence of diminished relational processing?. Journal of Autism and Developmental Disorders.

[CR9] Bowler DM, Gardiner JM, Berthollier N (2004). Source memory in Asperger’s syndrome. Journal of Autism and Developmental Disorders.

[CR10] Bowler DM, Gardiner JM, Gaigg SB (2007). Factors affecting conscious awareness in the recollective experience of adults with Asperger’s syndrome. Consciousness and Cognition.

[CR11] Bowler DM, Gardiner JM, Grice S (2000). Episodic memory and remembering in adults with Asperger’s syndrome. Journal of Autism and Developmental Disorders.

[CR12] Bowler DM, Matthews NJ, Gardiner JM (1997). Asperger’s syndrome and memory: Similarity to autism but not amnesia. Neuropsychologia.

[CR13] Cheung M, Chan AS, Sze SL, Leung WW (2010). Verbal memory deficits in relation to organization strategy in high- and low-functioning children. Research in Autism Spectrum Disorders.

[CR14] Coltheart M (1981). The MRC psycholinguistic database. Quarterly Journal of Experimental Psychology.

[CR15] Farrant A, Blades M, Boucher J (1998). Source monitoring in children with autism. Journal of Autism and Developmental Disorders.

[CR100] Gaigg SB, Bowler DM, Gardiner JM (2014). Episodic but not semantic order memory difficulties in autism spectrum disorder: evidence from the historical figures task. Memory.

[CR101] Kucera H, Francis WN (1967). Computational analysis of present-day American English.

[CR102] Lind SE, Bowler DM (2010). Episodic memory and episodic future thinking in adults with autism. Journal of Abnormal Psychology.

[CR103] Lind SE, Bowler DM, Crane L (2012). Remembering the past and imagining the future in autism spectrum disorder. Memory.

[CR16] Lord C, Rutter M, Goode S, Heemsbergen J, Jordan J, Mawhood L, Schopler E (1989). Autism Diagnostic Observation Schedule: A standardised observation of communicative and social behavior. Journal of Autism and Developmental Disorders.

[CR17] Martin JS, Poirier M, Bowler DM (2010). Brief report: Impaired temporal reproduction performance in adults with autism spectrum disorder. Journal of Autism and Developmental Disorders.

[CR18] Poirier M, Martin JS, Gaigg SB, Bowler DM (2011). Short term memory in autism spectrum disorder. Journal of Abnormal Psychology.

[CR19] Tulving E (2001). Episodic memory and common sense: How far apart?. Philosophical Transactions of the Royal Society, B.

[CR20] Williams, D., Boucher, J., Lind, S., & Jarrold, C. (2012). Time-based and event-based prospective memory in autism spectrum disorder: the roles of executive function, theory of mind and time estimation. *Journal of Autism and Developmental Disorders* (online first). 10.1007/s10803-012-1703-9 23179340

[CR21] Williams DL, Goldstein G, Minshew NJ (2006). The profile of memory function in children with autism. Neuropsychology.

